# Experimental Analysis of Gravitational Vortex Turbine Made from Natural Fibers

**DOI:** 10.3390/ma18102352

**Published:** 2025-05-19

**Authors:** María Varga, Laura Velásquez, Ainhoa Rubio-Clemente, Bladimir Ramón Valencia, Edwin Chica

**Affiliations:** 1 Grupo de Energía Alternativa, Facultad de Ingeniería, Universidad de Antioquia, Calle 70 No. 52-21, Medellín 050010, Colombia; mjose.vargas@udea.edu.co (M.V.); ainhoa.rubioc@udea.edu.co (A.R.-C.); edwin.chica@udea.edu.co (E.C.); 2 Escuela Ambiental, Facultad de Ingeniería, Universidad de Antioquia, Calle 70 No. 52-21, Medellín 050010, Colombia; 3 Grupo de Investigación de Ingeniería Mecánica de la Universidad de Pamplona-GIMUP, Facultad de Ingeniería y Arquitectur, Universidad de Pamplona, Km 1 Vía Bucaramanga Ciudad Universitaria, Pamplona 543050, Colombia; hbladimir@unipamplona.edu.co

**Keywords:** gravitational vortex turbine, renewable energy, natural fiber, PLA, 3D printing, energy efficiency

## Abstract

The use of natural fibers in hydro turbine rotors promotes sustainability by offering biodegradable, renewable materials with a lower carbon footprint. This study compares the hydrodynamic performance of two rotors in a gravitational vortex turbine: Rotor 1, 3D-printed with polylactic acid (PLA), and Rotor 2, made from fique fiber and epoxy resin using manual molding. To compare the rotors, experimental tests were conducted on a laboratory-scale setup, where the behavior of both rotors was evaluated under different flow regimes. Rotor 1 achieved 61.01% efficiency at an angular velocity (ω) 160 RPM, while Rotor 2 reached only 19.03% at ω of 165 RPM. The lower performance of Rotor 2 was due to dynamic imbalances and mechanical vibrations, leading to energy losses. These challenges highlight the limitations of manual molding in achieving precise rotor geometry and balance. To improve natural fiber rotor viability, optimizing manufacturing techniques is crucial to enhance dynamic balance and minimize vibrations. Advancements in fabrication could bridge the performance gap between natural and synthetic materials, making bio-based rotors more competitive. This study emphasizes the potential of natural fibers in sustainable energy and the need to refine production methods to maximize efficiency and reliability. Addressing these challenges will help integrate eco-friendly rotors into hydro turbine technologies.

## 1. Introduction

The rapid increase in global energy consumption has led to a strong dependence on fossil fuels, further exacerbating environmental issues such as climate change, air pollution, and ecosystem degradation [1,2]. As energy demand continues to rise, the urgency to diversify the energy matrix and invest in renewable sources becomes even more critical. However, many traditional solutions, such as large hydroelectric plants, require expensive infrastructure and can have negative environmental impacts, including the disruption of aquatic ecosystems and the displacement of communities [3,4]. In this scenario, it is essential to develop more accessible and sustainable technologies that allow for the efficient use of natural resources while minimizing ecological impact. Additionally, addressing these challenges requires innovative policies, improved energy storage solutions, and decentralized renewable energy systems to ensure a more resilient and environmentally friendly energy future.

Gravitational vortex turbines represent an alternative in the field of hydrokinetic energy generation, as they enable the transformation of water energy into electricity without the need for dams or reservoirs [5,6]. These turbines operate by creating a vortex within a circulation and discharge chamber, harnessing the kinetic energy of the water flow to drive a rotor connected to a generator. Their compact design and ability to function with low water flow rates make them an ideal solution for rural communities or regions with limited water resources. The power generation system consists of an inlet channel, a circulation chamber, a discharge chamber, the rotor, the electric generator, and the regulation and control system [7].

Despite advancements in the design and optimization of gravitational vortex turbines, most current studies have focused on improving their hydrodynamic and mechanical performance through both numerical and experimental research [8,9,10,11]. However, these developments have primarily relied on conventional materials such as metals and synthetic polymers, due to their favorable mechanical properties and compatibility with existing manufacturing processes. In laboratory-scale models, the use of 3D printing filaments and recycled polymers has been reported, which not only facilitates the fabrication of complex geometries with high precision but also promotes a more sustainable approach. In practical applications, steel and concrete structures have been employed, where steel allows for faster construction and greater recyclability, while reinforced concrete, though more cost-effective, requires extended curing times [5,12,13].

To date, only one study has been identified that combines fique natural fibers with gravitational vortex turbines. The authors of [14] evaluated a biocomposite material made from unsaturated polyester resin reinforced with fique fiber as a sustainable and cost-effective alternative for constructing rotor blades of a gravitational vortex turbine. Using the manual impregnation method, laminates with three layers of fique fiber were produced, yielding a lightweight material with promising mechanical properties. Compared to conventional materials like aluminum and stainless steel, the biocomposite demonstrated lower density and environmental advantages. Mechanical tests (tensile and bending) confirmed its suitability, and a functional rotor prototype was successfully manufactured, highlighting the biocomposite’s potential for application in hydrokinetic systems.

The use of materials such as biopolymers or natural fiber-reinforced composites presents a significant opportunity to reduce environmental impact and enhance the functionality of these systems. Integrating these materials into the development of gravitational vortex turbines could optimize their energy efficiency throughout their entire lifecycle, from production to end-of-life disposal. This opens new avenues for research in the field of sustainable materials, enabling the creation of more efficient and environmentally responsible technologies for renewable energy generation. Furthermore, the implementation of natural materials in the fabrication of turbine rotors could minimize their environmental footprint and promote the use of local resources, thereby contributing to a more sustainable and accessible energy transition. However, their implementation faces critical challenges, including inferior mechanical properties, susceptibility to moisture, complex manufacturing processes, and difficulties in achieving optimal dynamic balance. Additionally, the variability in the properties of natural materials and their potentially higher costs may limit their scalability and competitiveness. Overcoming these obstacles requires advances in material treatment, optimization of manufacturing processes, and the development of strategies for sustainable end-of-life management.

The manufacturing of rotors for gravitational vortex turbines using biopolymers and natural fibers can be approached through various processes, each with its own advantages and challenges. Three-dimensional printing stands out for its high precision and ability to create complex geometries, reducing material waste [15,16]; however, it may be limited by the mechanical strength of biopolymers and the costs associated with large-scale production. This process can be enhanced by incorporating natural fibers, such as fique, hemp, or flax, into biopolymer matrices like polylactic acid (PLA), enabling the creation of sustainable and customized components. Despite its potential, challenges such as the lower mechanical strength of natural fibers, the need to adjust printing parameters to avoid thermal degradation, and the higher costs of producing composite filaments must be addressed. On the other hand, compression molding is a scalable and efficient option for natural fiber-reinforced composites, although it requires high-precision molds that can increase initial costs and pose challenges in achieving optimal dynamic balance. Injection molding offers high repeatability and precision, making it ideal for uniform geometries, but it is restricted to materials with suitable flow properties and involves high equipment and mold costs. The manual molding process provides flexibility in using different fibers and resins, being low-cost for prototypes, but its precision and consistency are lower, which can result in dynamic imbalances. A hybrid approach, combining techniques such as 3D printing for critical parts and compression molding for final assembly, leverages the strengths of multiple processes, though it increases complexity and costs. Finally, post-manufacturing treatments, such as precision machining or the application of coatings, improve rotor quality and durability but add time and production costs. Overall, the choice of process will depend on factors such as production volume, required material properties, and available resources, making it crucial to explore innovative approaches that maximize the performance and sustainability of these materials. Integrating natural fibers into advanced manufacturing processes like 3D printing not only enhances sustainability but also opens new possibilities for creating efficient and eco-friendly energy systems.

This study presents the experimental characterization of a rotor for a gravitational vortex turbine fabricated using natural fibers, aiming to evaluate its performance and viability as an eco-friendly alternative for electric power generation. The importance and novelty of this article lie in the use of natural fibers as a manufacturing material, as the rotor design has already been validated previously. Key parameters such as system efficiency, structural resistance of the materials, and flow behavior are analyzed to assess its potential for applications in resource-limited areas. Additionally, the performance of the natural fiber rotor is compared to that of a 3D-printed rotor made from PLA, a biodegradable thermoplastic, providing insights into the advantages and limitations of each material in terms of energy generation and sustainability.

## 2. Materials and Methods

### 2.1. Importance of Natural Fibers in the Manufacturing of Turbine Rotors

The use of natural fibers in the manufacturing of hydro turbine rotors represents a significant step toward more sustainable energy solutions. Unlike synthetic materials such as fiberglass or carbon fiber, natural fibers such as flax, hemp, or jute are biodegradable, renewable, and have a lower carbon footprint. Their production requires less energy and generates fewer emissions, contributing to a more environmentally friendly manufacturing process. In addition, natural fiber composites offer a favorable strength-to-weight ratio, making them a competitive alternative for the reinforcement of modern composite materials [17]. Compared to synthetic fibers and fiberglass, they overcome various limitations by offering improved mechanical, thermal, and physical properties, which can enhance turbine efficiency while reducing mechanical stress on other components [18].

Furthermore, by minimizing reliance on petroleum-based materials, the adoption of natural fibers helps reduce environmental pollution and long-term waste accumulation. From a socio-economic perspective, incorporating locally sourced natural fibers supports rural economies and promotes sustainable agricultural practices, creating a circular economy model. Furthermore, these materials typically require fewer harmful chemicals during processing, lowering health risks for workers and reducing pollution in aquatic ecosystems. By integrating natural fibers into hydro turbine rotors, it is possible to achieve an optimal balance between performance, durability, and environmental responsibility, paving the way for more sustainable hydrokinetic energy systems.

A clear example of their implementation is the development of wind turbine blades made from composites based on sisal fibers, which not only reduces the carbon footprint of their production but also facilitates their recycling at the end of their useful life. Similarly, the automotive industry has begun to integrate natural fibers into the manufacture of structural components and interior panels, achieving lighter and more energy-efficient vehicles [19].

According to Amzil et al., the integration of hemp fibers into the manufacture of wind turbine blades has shown significant economic and environmental advantages, while maintaining established design requirements. These findings highlight their potential as a viable alternative to traditional glass fiber-reinforced composite materials. The studies indicate that this modification allowed for a reduction in both density and cost compared to a reference model composed exclusively of glass fiber and epoxy resin [20].

The study conducted by Rua et al. investigated the use of fique fibers as reinforcement in a composite material with epoxy resin, allowing for exploring new limits in the dynamic behavior of these materials. The results indicated that the composite material had lower stiffness compared to pure epoxy resin; but, at the same time, it showed better performance under stress conditions. In addition, its fracture was not brittle, evidencing greater flexibility and a better response to deformation under dynamic loading. Its impact resistance under static loading conditions was also highlighted [21].

In Colombia, the fique industry is widely recognized, with an annual production close to 30,000 tons, making this fiber an attractive alternative for the development of new composite materials. The viability and application of these composites depend largely on economic factors, where weight reduction, cost reduction, and the availability of sustainable raw materials play a key role. Based on these findings, fique fiber was selected as a reinforcing material in this research.

For the matrix, a polyester resin from the Poliescol brand was used. It is thermosetting with a density of 1.15 g/mL, has gel time of 6 to 9 min, has a viscous pink appearance, and the recommended hardener is methyl ethyl ketone peroxide (MEK peroxide) mixed with 1% by weight. The polyester resin has an elasticity modulus of 3.6 GPa, a tensile strength of 45 Mpa, and an elongation of 2.4%. The fique fiber, also called Furcraea Andina, is an endemic species native to the South American Andes, which has an asymmetric 0/90° braiding configuration, with double fiber in the warp direction. The fique has a modulus of 8.2 to 9.1 GPa, a tensile strength of 132.4 Mpa, and an elongation of 9.8%. This fiber was analyzed using various characterization techniques to evaluate its morphology, chemical structure, and thermal properties. Morphological analysis by scanning electron microscopy (SEM) reveals that fique fibers have a compact surface, without fibrillation, and are separated by approximately 10 µm. They present roughness, typical of raw natural fibers, with some impurities and waxy material. In its cross-section, cell walls formed by microfibrils and a central lumen are observed, with a wall thickness between 1 and 10 µm and a lumen diameter of 0.1 to 20 µm. Fique reflects an irregular and fibrous structure characteristic of natural fibers. In the analysis of fique fiber by Fourier Transform Infrared Spectroscopy (FTIR), the presence of hydroxyl groups, C-H in cellulose and hemicellulose, was observed. In addition, carbonyl groups (C=O) and lignin (C=C) confirmed the lignocellulosic composition of the fiber [22].

The characterization of the fique fiber composite with a polyester resin matrix focused on the interaction between the fiber and the polymeric matrix, evaluating its mechanical, thermal, and interface behavior. The morphological analysis of the fiber–matrix interface by SEM observed the interface between the fiber and the polyester resin. The fiber, with its rough surface and compact structure, shows a good interaction with the resin, which translates into greater compatibility and anchoring between both components. The resin seems to effectively impregnate the fique fibers, taking advantage of the surface roughness for better adhesion. In the mechanical properties, the results indicate that the composite presents an improvement in mechanical resistance compared to natural fibers without a matrix, thanks to the reinforcement provided by the polyester resin. The thermogravimetric analysis of the composites showed that the presence of polyester resin affects the thermal behavior of the material. The resin contributes to the thermal stabilization of the composite, although it may slightly alter the degradation temperature range compared to pure fique fibers. This type of composite, which combines natural fibers with polymeric matrices, presents a promising path towards sustainability in engineering materials, especially in industries seeking green solutions without compromising mechanical performance.

### 2.2. Geometry of the Gravitational Vortex Turbine Rotor

Gravitational vortex turbines are devices designed for hydrokinetic energy conversion through the generation of a stable vortex within a basin or vortex chamber. Their operating principle is based on inducing a rotational flow of water inside a cylindrical or conical tank, allowing the transformation of the fluid’s kinetic energy into mechanical energy via a rotor [23]. Water enters the vortex chamber through a tangential inlet, generating rotational movement within the fluid. The basin’s geometry influences the formation of a downward helical flow, characteristic of the gravitational vortex. A rotor, positioned at the center of this vortex, is driven by the motion of the water. The captured mechanical energy is transferred to an electric generator, enabling its conversion into usable power. These turbines exhibit high efficiency in low-flow and low-head environments, making them suitable for renewable energy generation in rivers and water bodies with limited hydraulic conditions [10].

The efficiency of gravitational vortex turbines is typically calculated by comparing the mechanical power output of the turbine ($P_{out}$) to the hydraulic power available in the flowing water (*P*). The hydraulic power is determined using the equation P=ρgQH, where ρ is the density of water, *g* is the acceleration due to gravity, *Q* is the volumetric flow rate, and *H* is the net head. The mechanical power output is usually obtained from measurements of torque (*T*) and rotational speed (ω). The overall efficiency (η) is then calculated using Equation (1) [8,10]:

(1) $$\eta =\frac{P_{out}}{P}=\frac{T\omega }{\rho gQH}$$

**Figure 1:**
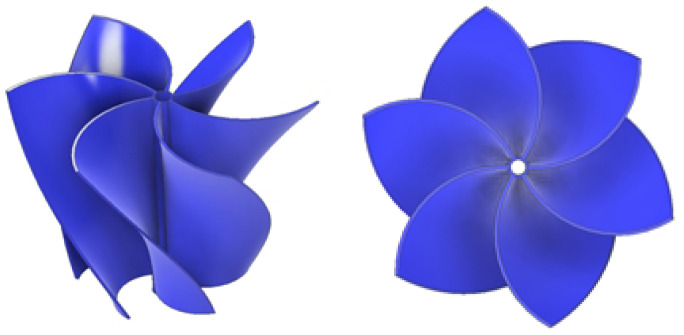
Rotor design.

Figure 1 presents the turbine under study, designed for efficient energy conversion from the gravitational vortex. The rotor, optimized by Velásquez et al. [8], features an advanced geometry that enhances performance by maximizing momentum transfer and minimizing energy losses. The curved blade configuration contributes to vortex stability, reinforcing its structure and optimizing turbine efficiency. Additionally, the blades are strategically inclined toward the rotor’s center, promoting vortex concentration in the central region, which enhances fluid interaction and improves energy conversion.

**Figure 2:**
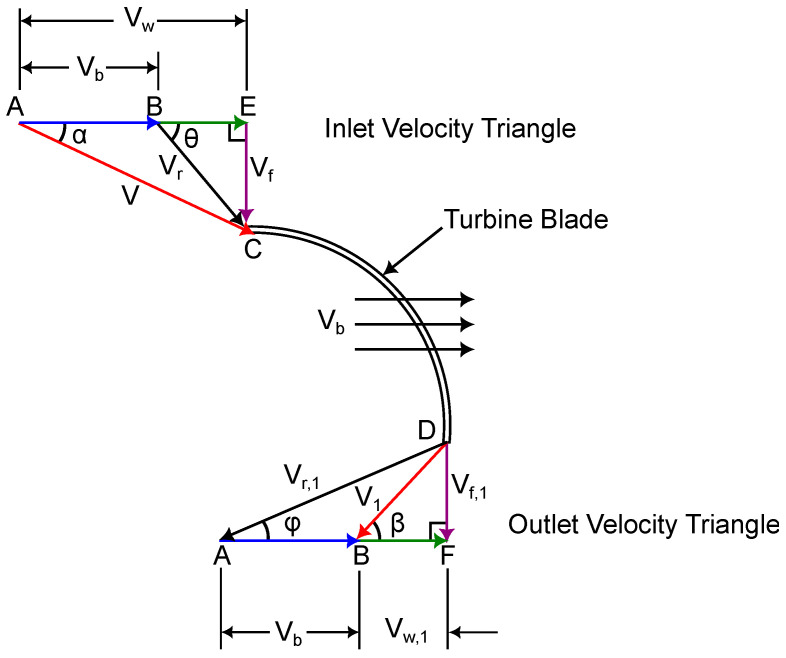
Velocity triangle.

The rotor design incorporates specific inlet and outlet angles that govern fluid interaction with the blades, directly influencing velocity and momentum transfer—see Figure 2. The blade inlet angle (α) is set at 16°, allowing water to enter smoothly while maintaining vortex stability, while the blade outlet angle (ϕ) is 90°, ensuring an efficient redirection of flow. Similarly, the absolute velocity inlet angle (θ) is 40°, and the absolute velocity outlet angle (β) is 43.8°, optimizing hydrodynamic performance by minimizing energy dissipation and improving momentum transfer to the rotor. The relative velocity twist angle (λ) of 68.8° further enhances fluid guidance along the blade surface, contributing to a stable and efficient flow pattern.

**Figure 3:**
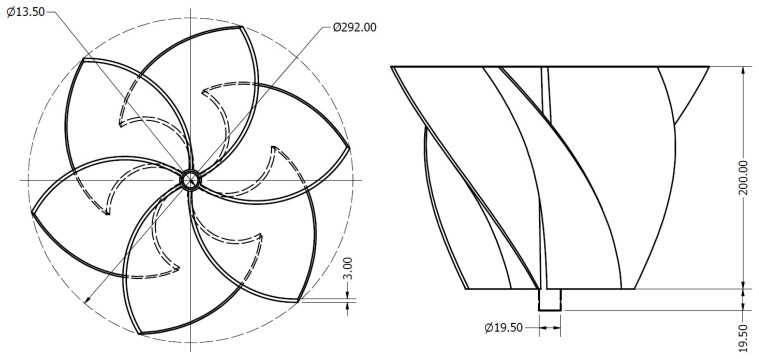
Dimension of the rotor.

Additionally, the rotor has a blade height (*L*) of 200 mm, a diameter of 292 mm, and consists of six blades, each with a thickness of 3 mm. These blades are joined to a central shaft with a diameter of 19.5 mm. Figure 3 and Table 1 present the main dimensions of the rotor under study. These geometric parameters influence vortex formation and the effective interaction between the fluid and the rotor, ensuring a balance between sustaining a strong vortex structure and maximizing energy extraction.

### 2.3. Manufacturing Process of Turbine Rotors Using 3D Printing

The manufacturing process of turbine rotors using 3D printing begins with the creation of a digital 3D model using computer-aided design (CAD) software Autodesk Inventor professional 2025, where the rotor’s geometry is optimized to meet performance criteria such as flow efficiency, strength, and durability. Once the design is finalized, an appropriate material is chosen for 3D printing, which may include thermoplastics, metal alloys, or composite filaments depending on the desired properties of the rotor, such as flexibility, strength, or resistance to corrosion. Using additive manufacturing, the rotor is then fabricated layer by layer, with the printer extruding or melting the chosen material through a nozzle to build the rotor’s structure from the bottom up. This method offers precise control over the geometry of the rotor, allowing for complex and detailed shapes that would be challenging to achieve through traditional manufacturing techniques [24,25].

**Figure 4:**
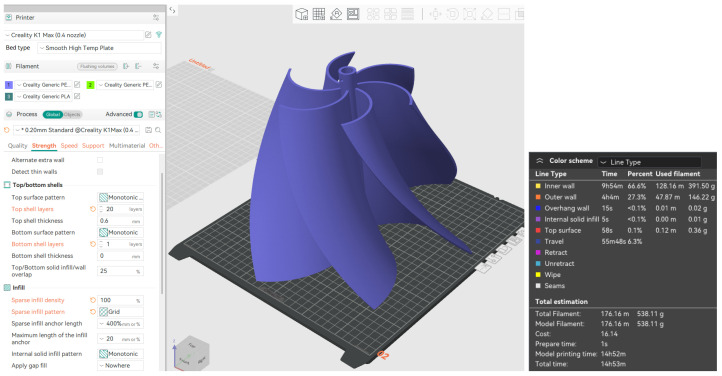
Printing parameters.

After printing, the rotor typically undergoes post-processing to enhance its surface finish and mechanical properties, including the removal of support structures, sanding, polishing, and curing the material for maximum strength. In the case of metal 3D printing, additional heat treatment may be used. Once the rotor is completed, it is assembled with other turbine components and tested under controlled conditions to assess its performance, mechanical strength, and efficiency. Based on the results, adjustments can be made to the design or manufacturing process to optimize the rotor’s performance, durability, and functionality. This process of 3D printing turbine rotors offers several advantages, such as reduced material waste, faster production times, and the ability to create highly customized and efficient designs for hydrokinetic and wind energy systems. The printing strategy of the rotor was first a 3D modeling in CAD software (Autodesk Inventor software 2025e) and then exported in STL format. PrusaSlicer was used to generate the G-code, optimizing printing parameters. The printing method was fused deposition molding (FDM), which is the standard technology in Ender 3 printers (Creality, Shenzhen, China). This method allows the rotor to be manufactured layer by layer, achieving a functional structure for initial tests. The rotor was printed using a PEGASUS Lite 3D printer (Creality, Shenzhen, China), which features a build volume of 300 × 300 × 300 mm. PLA was used for printing, with printing parameters with layer height 0.2 mm (for a balance between precision and printing time), 100% infill, printing speed 60 mm/s, extruder temperature 210 °C, bed temperature 60 °C (to improve adhesion), and support on the base. Figure 2 presents the printing parameters used in the software, as well as the total printing time and the amount of filament consumed. The total printing time was 14 h and 52 min, with 176.16 m of filament consumed, equivalent to 538.11 g. The printing parameters are presented in Figure 4.

### 2.4. Manufacturing Process of Natural Fiber Turbine Rotor

**Figure 5:**
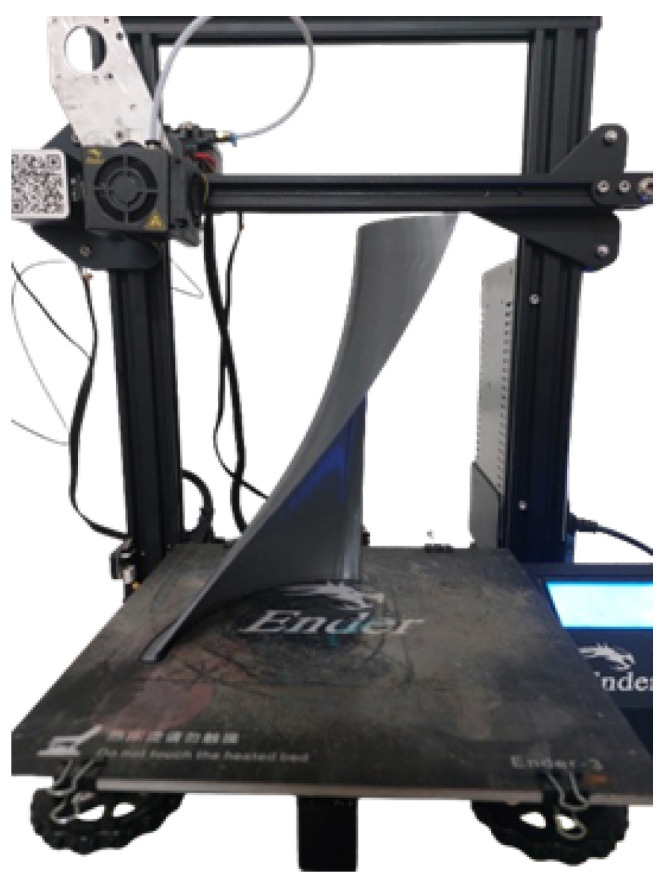
Model of the blade of the gravitational vortex turbine.

The manufacturing process for turbine rotors using natural fibers involves several key steps. First, the appropriate fique fiber material is selected and prepared. This preparation includes cleaning the selected fiber to remove impurities, ensuring that it will be suitable for integration into the composite material. Next, the natural fibers are combined with resins, either biocompatible or synthetic, to create a fiber composite material. The fibers are impregnated with resin using manual processes. The choice of resin depends on the performance needs of the rotor, with options including thermoplastic or thermosetting resins, which contribute to the mechanical strength of the material. Once the composite is prepared, it is molded into the desired blade shape using vacuum infusion, die casting, or hand lamination techniques. During molding, the resin hardens, providing structural integrity and strength to the composite. Once the blade is molded, it undergoes a curing process to ensure that the resin hardens completely, solidifying the fibers and allowing the rotor to meet the required mechanical properties. Once cured, the blades undergo finishing processes, which involve smoothing the surfaces and removing imperfections. The blades are then assembled onto the central shaft of the rotor, ensuring proper alignment and secure attachment. Finally, the rotor is tested under simulated operating conditions to assess its energy efficiency. The rotor is also checked to ensure it meets dynamic balancing and safety standards. During this stage, any necessary adjustments are made to optimize the rotor’s performance. The use of natural fibers in hydroelectric turbine rotors not only improves the sustainability of the energy system, but also offers better performance and durability, contributing to the development of more environmentally friendly hydrokinetic energy solutions. The process began with the design of the blades using Autodesk Inventor software, where geometric precision was pursued to meet hydraulic efficiency and structural stability requirements, such as blade thickness, among others. The rotor was modeled with six symmetrical blades with specific curvatures that maximize the energy conversion of the gravitational flow. The CAD model incorporated key geometric details, such as the aerodynamic profile and the length of the blades, which are decisive for the performance of the runner. Once the optimal design was finalized, only one blade was left and exported in high-resolution STL (Stereolithography) format for use in subsequent stages. The base model was then 3D printed using PLA filament. Printing was accomplished layer by layer, adjusting parameters such as nozzle temperature of 210 °C and layer speed of 30 mm/s, to obtain an accurate reproduction of the original design. The printed model, as seen in Figure 5, served as a template to replicate the geometry of the blades, taking advantage of the properties of PLA to capture fine details and ensure accuracy in the following stages.

**Figure 6:**
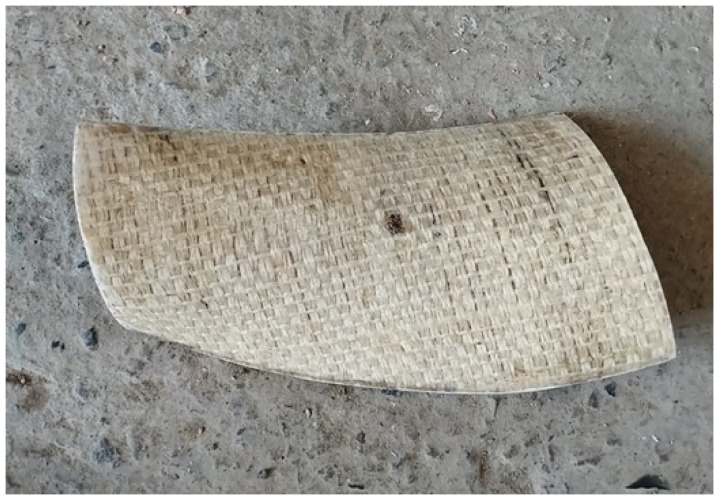
Blade manufactured with good finishes.

The next step was to manufacture the mold from the printed model. To achieve this, the piece was placed on a support structure and a mixture of polyester resin and fiberglass was applied. A fiberglass cloth of suitable size was arranged to cover the model and was subsequently impregnated with resin using a brush, ensuring uniform coverage. Once the material had solidified, the base model was removed, obtaining a precise cavity that reproduced the shape of the blade. For the manufacture of the blades, a combination of polyester resin and two layers of fique fibers as reinforcement supplied were used. The fibers were previously cut and prepared to fit the mold. Each layer was manually impregnated with polyester resin, guaranteeing complete saturation and the elimination of air bubbles. The layers were arranged following a weave pattern to optimize mechanical strength and structural uniformity. After lamination, the piece was left to cure at room temperature according to the resin manufacturer’s specifications. After curing, the blades were carefully removed to avoid damage to the edges and subjected to a manual polishing process with fine-grained abrasives as shown in Figure 6; this improved the surface quality and aerodynamics of the parts. Additionally, individual weight control was performed to ensure uniformity, a crucial aspect to prevent imbalances and vibrations during operation. In cases where weight variations were detected, adjustments were made by fine sanding until a homogeneous distribution of mass was achieved in all the parts.

**Figure 7:**
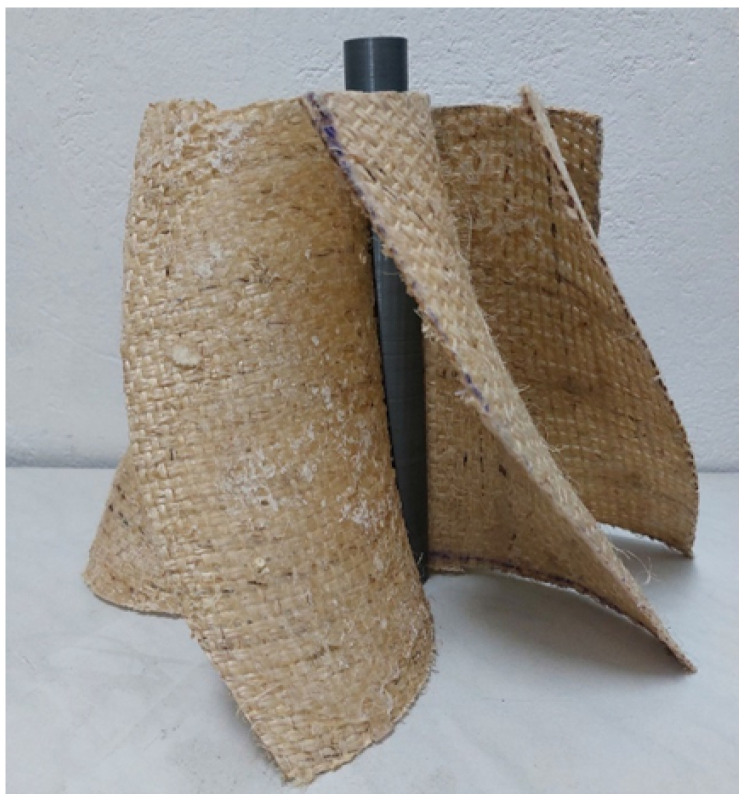
Fitting the blades to the shaft mold.

The central shaft was manufactured exclusively from polyester resin, molded into a cylindrical structure that provided the necessary rigidity and durability. For the assembly of the impeller, a special support was designed using 3D printing in PLA as seen in Figure 7, which allowed the blades to be precisely aligned around the shaft and maintain their position during the joining process.

**Figure 8:**
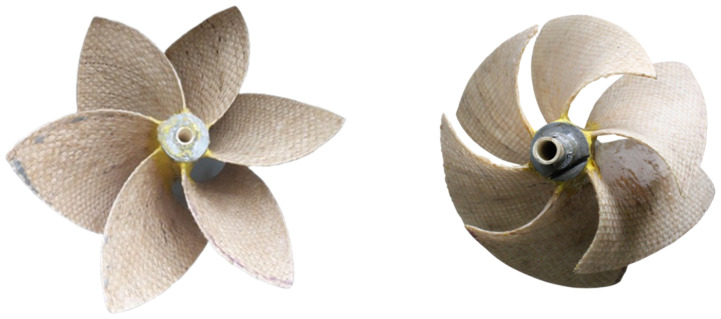
Final gravitational vortex turbine rotor.

Finally, the blades were assembled and fixed to the shaft made of polyester resin, ensuring a firm and stable connection. The assembly was then left to cure completely to ensure optimal adhesion between the components and improve structural strength. The result was a solid and durable connection, as seen in Figure 8.

The use of natural fibers such as fique in the construction of turbine blades presents several limitations. One of the main challenges is the natural variability of the fibers, as their properties can vary significantly depending on their origin and the treatments they undergo, which affects the reproducibility of the rotor. Additionally, while fique fibers have good tensile strength, they exhibit limited resistance to fatigue and erosion. In conditions of turbulent flow, erosion, or exposure to moisture, the fibers tend to degrade more quickly, reducing their durability. Furthermore, the high moisture absorption of fique fibers can compromise the structural integrity of the turbine. Moisture absorption can degrade the matrix or induce swelling, leading to a reduction in the dimensional and mechanical stability of the blades. Finally, fique composites tend to have a higher surface roughness compared to other materials if appropriate compaction techniques are not employed, which may negatively impact the hydraulic efficiency of the rotor.

### 2.5. Setup and Experimental Configuration for Rotor Testing

**Figure 9:**
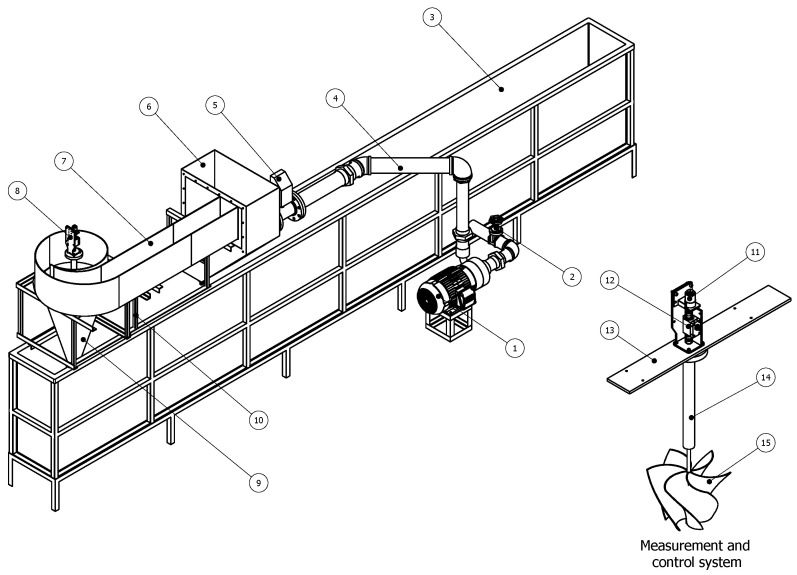
Experimental bench: (1) Centrifugal pump, (2) flow control system, (3) lower tank, (4) supply pipe, (5) flow meter, (6) feeding tank, (7) spiral inlet channel, (8) measurement and control system, (9) discharge cone, (10) support structure, (11) loading motor, (12) torque sensor, (13) adjustable support, (14) vertical shaft, and (15) rotor.

The test bench shown in Figure 9 is designed to evaluate the performance of a gravitational vortex turbine. The test bench consists of several components. The pumping system (1) includes an electric motor coupled to a centrifugal pump that generates the required water flow to feed the test channel. A flow control system (2) is installed at the pump outlet to regulate water velocity before entering the test section. The lower tank (3) collects the water after passing through the turbine, allowing its recirculation. The supply pipe (4) transports water from the lower tank to the feeding tank (6), where the flow stabilizes before entering the test section. A flow meter (5) measures the water flow entering the system, ensuring precise control of the test conditions. The spiral inlet channel (7) directs water toward the turbine, creating a vortex flow that enhances the gravitational vortex effect. The measurement and control system (8) records key parameters such as torque and rotational speed. The discharge cone (9) facilitates water exit from the turbine, ensuring proper flow toward the lower tank while minimizing unwanted turbulence. The support structure (10) holds the feeding system and ensures the stability of the test channel. A loading motor (11) is coupled to the turbine shaft to apply a controlled mechanical load during the tests, while a torque sensor (12) measures the torque generated by the turbine. The adjustable support (13) allows modifications to the position of the measurement and control system to accommodate different experimental configurations. A vertical shaft (14) connects the turbine rotor to the measurement and control system, transmitting rotational motion. Finally, the gravitational vortex turbine (15) is submerged at the bottom of the system, harnessing the vortex-induced flow to convert water energy into mechanical energy.

The experimental tests of the gravitational vortex turbine in the described test system follow a structured procedure. First, a specific flow rate is set, causing the fluid to rise through the pipe and fill the feeding tank. Once the tank reaches a certain level, water flows into the inlet channel, where it moves towards the discharge cone. Inside the cone, a water vortex forms, driving the rotation of the turbine. The measurement and control system records the turbine’s rotational velocity and torque. The sensor, through a connection to a computer, provides real-time values of torque and rotational speed under the different testing conditions.

Next, the motor coupled to the control system is powered, applying a load to the rotor and gradually slowing its rotation. As the current and voltage supplied to the rotor increase, the load intensifies, further reducing the rotor’s speed. This process is repeated incrementally until the rotor comes to a complete stop. The procedure is conducted multiple times to obtain the turbine’s performance curve. The tests were performed at two flow rates, 2.5 L/s and 3.0 L/s, and for two different rotors: Rotor 1, made of PLA, and Rotor 2, made of natural fiber.

Possible sources of measurement errors in the experimental setup include sensor inaccuracies, signal noise, mechanical misalignments, and flow instabilities. Torque and rotational speed measurements may be affected by slight miscalibrations of the sensor or variations in the connection between the rotor and the measuring shaft. Additionally, fluctuations in the flow rate due to turbulence or inconsistent valve regulation can introduce variability in the turbine’s performance data. To minimize these errors, all sensors were calibrated prior to testing, and measurements were taken multiple times to ensure repeatability. The flow was regulated using a constant head in the feeding tank to reduce instabilities, and the mechanical components were carefully aligned and checked for looseness or asymmetry. Data were recorded in real time and averaged over short intervals to smooth out transient variations and improve the reliability of the results.

## 3. Results and Discussion

### 3.1. Flow Visualization and Vortex–Rotor Interaction

**Figure 10:**
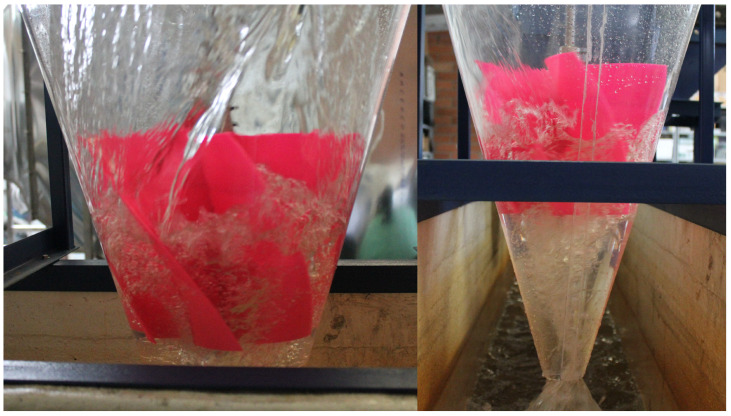
Vortex formation around Rotor 1 in a gravitational vortex turbine.

**Figure 11:**
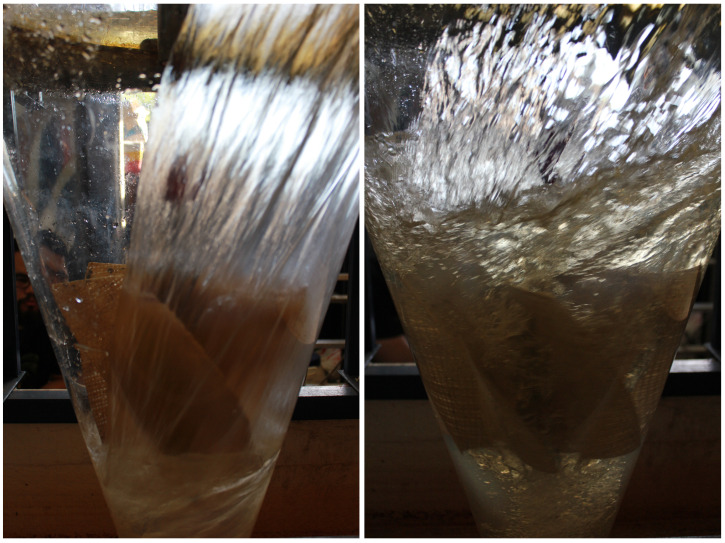
Vortex formation around Rotor 2 in a gravitational vortex turbine.

Figure 10 and Figure 11 present Rotors 1 and 2 operating inside the cone, highlighting the interaction between the gravitational vortex and the rotors. The rotors are partially submerged in the swirling water flow. The vortex forms a well-defined central core, with high-speed water spiraling downwards around the rotor. The transparency of the water surface allows for the visualization of flow patterns and turbulence generated by the rotating fluid. At first glance, the vortex behavior around both rotors seems similar, suggesting that the general flow structure is maintained regardless of the rotor material. However, further analysis would be required to determine subtle differences in energy extraction and efficiency between the two configurations.

### 3.2. Experimental Performance

**Figure 12:**
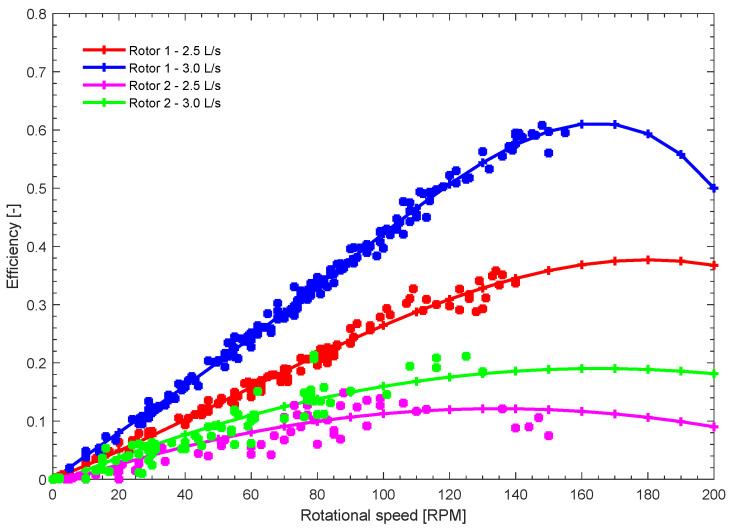
Experimental results.

Figure 12 presents the experimental performance for both rotors, showing the relationship between angular velocity and efficiency for the tested flow rates of 2.5 L/s and 3.0 L/s. The curves illustrate how efficiency varies with increasing angular velocity for each rotor and operating condition. Efficiency was calculated using Equation (1), based on the torque and rotational speed values provided by the measurement and control system. For both tests, the net head *H* used in the calculations was 42.2 cm.

Rotor 1, made of PLA, exhibits superior performance compared to Rotor 2, made of natural fiber, under both flow rate conditions. At 2.5 L/s, Rotor 1 achieves a maximum efficiency of approximately 0.35 at around 140 RPM. When the flow rate increases to 3.0 L/s, its performance improves significantly, reaching a peak efficiency close to 0.65 at approximately 160 RPM. In contrast, Rotor 2 demonstrates lower efficiency values across all conditions. At 2.5 L/s, its efficiency remains below 0.15, with a maximum reached near 120 RPM. When the flow rate increases to 3.0 L/s, the efficiency improves slightly but remains under 0.20, peaking around 140 RPM.

### 3.3. Impact of Manufacturing Quality on Performance

The natural fiber rotor (920 g) is significantly heavier than the PLA rotor (538.11 g). This difference affects its moment of inertia, meaning that more energy is required to start and stop it. A heavier rotor may take longer to reach its optimal speed, which could be reflected in performance curves with a smoother slope at the beginning. When increasing the load on the control system, the heavier rotor may better resist deceleration due to its higher inertia. However, if the material’s efficiency is low, it may also generate less power. The lower efficiency of the natural fiber rotor may be attributed to its greater weight, which implies higher resistance to motion. If the natural fiber rotor has higher inertia, it may require a stronger flow to achieve the same rotational speed as the PLA rotor. Additionally, the PLA rotor was fabricated in 14 h, whereas the natural fiber rotor required three days for production. This disparity in manufacturing time could result in variations in surface finish and geometric precision, ultimately influencing hydrodynamic efficiency. A rougher surface finish on the natural fiber rotor, potentially attributable to the fabrication process, may induce increased frictional drag, thereby diminishing its hydrodynamic performance. Conversely, the smooth surface of the PLA rotor can mitigate frictional forces and enhance fluid flow characteristics, contributing to improved efficiency. Furthermore, the dimensional accuracy afforded by additive manufacturing techniques, such as 3D printing, facilitates the production of more uniform and optimized rotor geometries, enhancing overall performance [26]. In contrast, the increased surface roughness of the fique fiber rotor may promote turbulence and energy dissipation, resulting in reduced efficiency. Moreover, the inherent variability in the fiber’s morphology can lead to inconsistencies in performance metrics. These differences in weight, inertia, and surface characteristics highlight the complex interplay between material properties and rotor performance.

### 3.4. Dynamic Balance of the Rotor

Beyond these factors, another crucial aspect influencing the efficiency of gravitational vortex turbines is the dynamic balance of the rotor. Regardless of the material used, an unbalanced rotor can introduce additional mechanical losses, vibrations, and structural stresses that further hinder energy conversion. The imbalance of a rotor is inherent such that the rotor’s rotation axis is not coincident with the geometric axis, resulting in rotational inertial force motivated when rotating [27]. Therefore, ensuring proper rotor balancing is essential for optimizing performance and maintaining the long-term stability of the system.

The importance of having a dynamically balanced rotor in gravitational vortex turbines lies in its direct impact on the efficiency and stability of the energy capture process. An unbalanced rotor generates vibrations and uneven forces that can negatively affect system performance, increasing mechanical losses and reducing the efficient conversion of water’s kinetic energy into usable power [28,29]. Proper rotor balancing minimizes unwanted oscillations that could compromise the turbine’s structural integrity and accelerate the wear of mechanical components [27]. Additionally, it reduces hydrodynamic resistance and improves operational stability, allowing the blades to maintain an optimal angle of attack relative to the water flow. This results in higher energy extraction efficiency, lower maintenance requirements, and an extended system lifespan. In the context of gravitational vortex turbines, where flow velocities can vary and generate fluctuating dynamic loads, a balanced rotor also enhances the turbine’s reliability. This is particularly crucial in remote applications or decentralized energy systems, where maintenance access may be limited. Therefore, ensuring precise dynamic balancing of the rotor not only optimizes energy efficiency but also enhances the sustainability and economic viability of gravitational vortex turbines, guaranteeing stable and long-term operation in various aquatic environments. In the case of gravitational vortex turbines, the manufacturing process of Rotor 2 did not ensure proper dynamic balancing, which may have led to vibrations and negatively impacted its energy conversion performance. As a result, before conducting experimental tests, it is necessary to perform a dynamic balancing process to correct any existing imbalance. However, this correction entails additional costs in both manufacturing and assembly, potentially affecting the economic feasibility of the design. This aspect was overlooked in the original study, suggesting that the differences observed in efficiency when comparing the two rotors may be directly attributed to the lack of proper balancing in Rotor 2. Therefore, future research should incorporate a more detailed analysis of dynamic balancing as a critical factor in the design and manufacturing of gravitational vortex turbine rotors.

The dynamic balancing of a rotor made from natural fibers using a manual molding process requires a systematic approach to minimize vibrations and optimize performance. First, an initial static balancing is performed by placing the rotor on a low-friction support to identify and correct any significant mass asymmetries by adding or removing material [30]. Next, for dynamic balancing, the rotor is mounted on a test rig equipped with vibration sensors and a high-speed rotational system. By gradually increasing the rotation speed, an accelerometer or laser vibrometer is used to detect imbalance-induced vibrations. Based on the collected data, small counterweights or resin adjustments are strategically applied to the rotor until the vibrations are minimized [27,31].

Given that the manual molding process may introduce inconsistencies in material distribution, achieving a precise balance is essential to enhance the turbine’s efficiency, reduce mechanical wear, and ensure long-term operational stability. Additionally, the manual molding process presents several disadvantages that can affect the quality and performance of the rotor in a gravitational vortex turbine. One of the main limitations is the difficulty in precisely controlling the applied material, leading to variations in blade thickness and uniformity. Factors such as applied pressure, resin distribution, and fiber arrangement may vary in each fabrication, making it challenging to achieve consistent rotor geometry. These inconsistencies can negatively impact the rotor’s aerodynamic and hydrodynamic properties, affecting its dynamic balance and overall energy conversion efficiency [32], especially when compared to automated manufacturing methods like injection molding or vacuum infusion.

To address this issue, several strategies can be implemented:

- Use of high-precision rigid molds: Constructing molds from aluminum or reinforced polymers to ensure better dimensional stability in the blades.
- Application of compaction techniques: Utilizing rollers or vacuum systems during resin curing to achieve a more uniform material distribution and prevent thickness inconsistencies.
- Thickness quality control: Inspecting components with calipers or 3D scanners to detect variations and make necessary corrections before dynamic balancing.
- Post-processing through machining or sanding: Manually adjusting the final blade thickness after fabrication to improve geometric precision and minimize imbalances. As part of future research, these measures will be implemented to refine the fabrication process and improve the performance of the gravitational vortex turbine rotors. By addressing these manufacturing challenges, the study aims to enhance the efficiency, durability, and feasibility of using natural fiber-based rotors in hydrokinetic applications.

## 4. Conclusions

The manufacturing of the rotor of the gravitational vortex turbine using composite materials and natural fibers represents a significant advance in engineering, demonstrating that it is possible to develop new sustainable materials applicable to hydraulic turbines. This study marks a first step towards optimizing the manufacturing process, allowing for improving rotor efficiency, optimizing material use, and reducing costs compared to traditional methods. Rotor 1, made of PLA, outperforms Rotor 2, made of natural fiber, in terms of efficiency. At a flow rate of 3.0 L/s, Rotor 1 achieves a peak efficiency of approximately 0.65 at around 160 RPM. In contrast, under the same flow conditions, Rotor 2 exhibits significantly lower efficiency, remaining below 0.20 and peaking at approximately 140 RPM. While the efficiency metrics of fique fiber rotors may show a performance deficit compared to their PLA counterparts, their inherent material properties offer distinct advantages in specific operational contexts. In such scenarios, the trade-off between efficiency and durability favors the use of fique fiber, ensuring prolonged and reliable performance in challenging environments. The selection of the natural fiber-based biocomposite was presented as a viable alternative for rotor manufacturing. Furthermore, proper curing of the polyester resin shaft guaranteed a firm and stable connection between the blades and the shaft, contributing to the structural durability of the assembly. Reinforcement with natural fibers allowed a significant reduction in weight compared to conventional materials, while maintaining adequate mechanical performance. This feature is especially beneficial in renewable energy applications, where structural efficiency is a key factor.

Nevertheless, several limitations and challenges were encountered during this study. The use of the manual rolling method, while accessible and cost-effective, posed significant difficulties in achieving uniform surface quality and consistent adhesion between layers, which are critical for maintaining a homogeneous blade thickness. Additionally, ensuring dynamic balance in the rotor proved challenging, directly affecting the turbine’s operational performance and lifespan. Vibrations resulting from imbalance not only reduced efficiency but also risked inducing structural damage over time. The handmade manufacturing process also introduced variability in the rotor dimensions, influencing the reproducibility of results. Future research should explore alternative fabrication techniques, such as vacuum infusion, to enhance the mechanical properties, surface finish, and durability of the rotors made with natural fibers. Additionally, optimizing fiber orientation and resin curing parameters could further improve structural integrity and overall turbine performance.

## Figures and Tables

**Table 1:** Main geometric parameters of the rotor.

Symbol	Variable	Value
α	Blade inlet angle [Deg]	16.00
ϕ	Blade outlet angle [Deg]	90.00
θ	Water inlet angle [Deg]	40.00
β	Water outlet angle [Deg]	43.80
λ	Twist angle [Deg]	68.80
*L*	Blade height [mm]	200.00
*D*	Rotor diameter [mm]	292.00
*n*	Number of blades	6.00
*t*	Blade thickness [mm]	3.00

## Data Availability

The original contributions presented in this study are included in the article. Further inquiries can be directed to the corresponding author.

## Abbreviations

PLA	Polylactic acid
MEK	Methyl ethyl ketone
SEM	Scanning electron microscopy
FTIR	Fourier transform infrared
CAD	Computer-aided design
FDM	Fused deposition molding
STL	Stereolithography
$P_{out}$	Mechanical power output
*P*	Hydraulic power
ρ	Density of water
*g*	Gravity
*Q*	Volumetric flow rate
*H*	Net head
*T*	Torque
η	Efficiency
ω	Angular velocity
α	Blade inlet angle
ϕ	Blade outlet angle
θ	Absolute velocity inlet angle
β	Absolute velocity outlet angle
λ	Relative velocity twist angle
*L*	Blade height
*D*	Rotor diameter
*n*	Number of blade
*t*	Blade thickness
